# Blood–brain barrier leakage and perivascular inflammation in cerebral amyloid angiopathy

**DOI:** 10.1093/braincomms/fcac245

**Published:** 2022-09-26

**Authors:** Mariel G Kozberg, Irvin Yi, Whitney M Freeze, Corinne A Auger, Ashley A Scherlek, Steven M Greenberg, Susanne J van Veluw

**Affiliations:** MassGeneral Institute for Neurodegenerative Disease, Massachusetts General Hospital/Harvard Medical School, Boston, MA 02129, USA; J. Philip Kistler Stroke Research Center, Massachusetts General Hospital/Harvard Medical School, Boston, MA 02114, USA; Department of Economics, Harvard College, Harvard University, Cambridge, MA 02138, USA; Department of Radiology, Leiden University Medical Center, Leiden, 2333 ZD, The Netherlands; Department of Neuropsychology and Psychiatry, Maastricht University, Maastricht, 6200 MD, The Netherlands; MassGeneral Institute for Neurodegenerative Disease, Massachusetts General Hospital/Harvard Medical School, Boston, MA 02129, USA; MassGeneral Institute for Neurodegenerative Disease, Massachusetts General Hospital/Harvard Medical School, Boston, MA 02129, USA; J. Philip Kistler Stroke Research Center, Massachusetts General Hospital/Harvard Medical School, Boston, MA 02114, USA; MassGeneral Institute for Neurodegenerative Disease, Massachusetts General Hospital/Harvard Medical School, Boston, MA 02129, USA; J. Philip Kistler Stroke Research Center, Massachusetts General Hospital/Harvard Medical School, Boston, MA 02114, USA; Department of Radiology, Leiden University Medical Center, Leiden, 2333 ZD, The Netherlands

**Keywords:** cerebral amyloid angiopathy, microbleeds, perivascular inflammation, blood–brain barrier leakage

## Abstract

Cerebral amyloid angiopathy is a small vessel disease associated with cortical microbleeds and lobar intracerebral haemorrhage due to amyloid-β deposition in the walls of leptomeningeal and cortical arterioles. The mechanisms of cerebral amyloid angiopathy–related haemorrhage remain largely unknown. Recent work has demonstrated that ruptured blood vessels have limited (or no) amyloid-β at the site of bleeding and evidence of local vascular remodelling. We hypothesized that blood–brain barrier leakage and perivascular inflammation may be involved in this remodelling process. This study examined cortical arterioles at various stages of cerebral amyloid angiopathy–related vascular pathology (without evidence of microhaemorrhage) in autopsy tissue from seven cases with definite cerebral amyloid angiopathy. We included temporo-occipital sections with microbleeds guided by *ex vivo* MRI from two cases with severe cerebral amyloid angiopathy and systematically sampled occipital sections from five consecutive cases with varying cerebral amyloid angiopathy severity. Haematoxylin and eosin stains and immunohistochemistry against amyloid-β, fibrin(ogen), smooth muscle actin, reactive astrocytes (glial fibrillary acidic protein) and activated microglia (cluster of differentiation 68) were performed. Arterioles were graded using a previously proposed scale of individual vessel cerebral amyloid angiopathy severity, and a blinded assessment for blood–brain barrier leakage, smooth muscle actin and perivascular inflammation was performed. Blood–brain barrier leakage and smooth muscle actin loss were observed in significantly more vessels with mild amyloid-β deposition (Grade 1 vessels; *P* = 0.044 and *P* = 0.012, respectively) as compared to vessels with no amyloid-β (Grade 0), and blood–brain barrier leakage was observed in 100% of vessels with evidence of vessel remodelling (Grades 3 and 4). Perivascular inflammation in the form of reactive astrocytes and activated microglia was observed predominantly surrounding arterioles at later stages of vessel pathology (Grades 2–4) and consistently around vessels with the same morphological features as ruptured vessel segments (Grade 4). These findings suggest a role for blood–brain barrier leakage and perivascular inflammation leading to arteriolar remodelling and haemorrhage in cerebral amyloid angiopathy, with early blood–brain barrier leakage as a potential trigger for subsequent perivascular inflammation.

## Teaser

Kozberg *et al*. examined blood–brain barrier leakage and perivascular inflammation at the individual vessel level in cerebral amyloid angiopathy. They report blood–brain barrier leakage at early stages of vascular pathology, a potential trigger for perivascular inflammation and vascular remodelling leading to haemorrhage.

## Graphical Abstract

**Graphical abstract:**
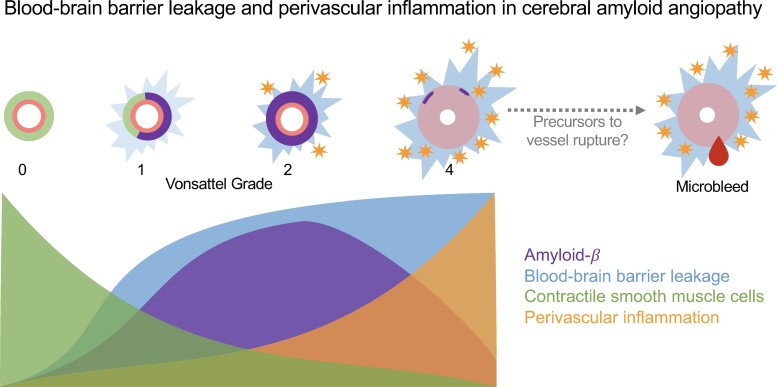

## Introduction

Cerebral amyloid angiopathy (CAA) is characterized by the deposition of amyloid-β (Aβ) in the walls of cortical and leptomeningeal arterioles in the brain.^1^ CAA is a leading cause of symptomatic intracerebral haemorrhage (ICH) in the elderly and is also associated with smaller haemorrhagic lesions including lobar cerebral microbleeds (CMBs) and cortical superficial siderosis (cSS).^2^

The mechanisms leading to haemorrhage in CAA remain largely unknown. Direct damage from Aβ deposition has been hypothesized to lead to vessel fragility and rupture; however, recent work examining CMBs has demonstrated decreased or no Aβ as well as vessel remodelling in the form of fibrinoid necrosis at the site of vessel rupture.^3,4^ These remodelled arterioles have evidence of blood–brain barrier (BBB) leakage in the form of fibrin positivity in vessel walls and surrounding tissue. A recent study reported a positive association between the percentage of fibrin-positive vessels and the number of CMBs in CAA.^5^ Additionally, the presence of remodelled vessels with decreased Aβ and fibrinoid necrosis is strongly associated with symptomatic ICH risk.^6^ These associations with haemorrhage are in fact stronger than the associations between Aβ deposition and haemorrhage,^5^ suggesting that other pathophysiological mechanisms besides Aβ deposition alone play a role in the cascade of events leading up to haemorrhage in CAA.

A subset of patients with CAA develops spontaneous episodes of focal, severe inflammation leading to clinical symptoms including subacute cognitive decline, focal neurological deficits, headaches and seizures. These episodes are termed CAA-related inflammation (CAA-ri).^7–9^ In these cases, regions of inflammation have an increased CMB burden, suggesting that active inflammation may contribute to haemorrhage.^10^ Additionally, Pittsburgh compound B positron emission tomography imaging studies suggest that CAA-ri may involve Aβ removal from the brain and anti-Aβ autoantibodies have been identified in the cerebrospinal fluid of patients with active CAA-ri.^11,12^ Anti-Aβ immunotherapies for Alzheimer’s disease, which rely on immune-mediated Aβ removal from the brain, are associated with adverse events termed amyloid-related imaging abnormalities (ARIAs).^13^ These events involve both local inflammation with vasogenic oedema (ARIA-E) and haemorrhage (ARIA-H), and CAA has been proposed as an important contributing factor in the development of ARIA.^13,14^ Similar to episodes of CAA-ri, haemorrhages (including CMBs and cSS) in ARIA-H typically colocalize with regions of inflammation and oedema.^13^

We hypothesize that vascular remodelling and subsequent CMBs in sporadic CAA may be in part driven by local inflammation, in a more subtle way than in CAA-ri or ARIA. To further understand the relationships between BBB leakage, perivascular inflammation and vessel wall integrity in vessels at risk for haemorrhage, we investigated each at the level of individual unruptured arterioles. We performed a detailed neuropathological evaluation of serial sections of post-mortem brain tissue from patients with definite CAA, aided by *ex vivo* MRI. Cortical arterioles were graded using the Greenberg and Vonsattel score of vascular CAA severity^15^ and assessed for markers of BBB leakage, reactive astrocytes, activated microglia and contractile smooth muscle cell integrity.

## Materials and methods

### Post-mortem MRI acquisition and analysis

Seven intact formalin-fixed human brain hemispheres were received through an ongoing brain donation programme at Massachusetts General Hospital (MGH). These CAA cases were patients diagnosed with possible or probable CAA during life, and diagnosis was confirmed through medical records and available clinical MRI or CT during life, as well as upon autopsy (see Table 1). Study approval was received from the MGH institutional review board and informed consent was obtained from the next of kin or another legal representative prior to autopsy.

**Table 1:** Case characteristics

Case #	Sex	Age at death (years)	Cause of death	Cortical CAA Burden score^a^	MRI CMBs count^b^	ABC score^c^
Targeted cases						
1	M	70	ICH	9	261	A3B3C1
2	F	79	ICH	8	204	A3B3C2
Consecutive cases						
3	M	67	ICH	12	168	A3B1C2
4	M	76	Unknown	11	31	A3B3C3
5	F	78	Unknown	7	17	A3B2C1
6	M	86	Unknown	10	9	A3B3C3
7	M	85	Unknown	12	49	A3B3C3
ARIA-E case						
8	F	74	Pulmonary embolism	Not assessed	Not available	A3B3C3

^a^ CAA severity was evaluated on Aβ-stained sections from predefined regions of frontal, temporal, parietal and occipital cortices using a 4-point scale (0, absent; 1, scant Aβ deposition; 2, some circumferential Aβ; 3, widespread circumferential Aβ) following proposed consensus criteria.^16^ Scores from the four areas were added to form a cumulative CAA severity score. ^b^Total number of CMBs across each sampled hemisphere was assessed on *ex vivo* 3T MRI by an experienced rater (S.J.v.V.).^3^^c^ABC scores were taken from routine neuropathological examination reports and reflect the National Institute on Aging-Alzheimer’s Association score for Alzheimer’s disease neuropathologic changes.^17^

High-resolution *ex vivo* 3T MRI was utilized to detect CMBs. Each hemisphere was prepared and scanned as previously described.^3^ Briefly, the hemispheres were packed in a plastic bag filled with periodate-lysine-paraformaldehyde fixative and vacuum sealed to remove air bubbles. The hemispheres were scanned overnight using a 32-channel head coil and a whole-body 3T MRI scanner (MAGNETOM Trio; Siemens Healthineers, Erlangen, Germany). The protocol included a T_2_-weighted turbo-spin echo (TSE) sequence (resolution = 500 × 500 × 500 μm^3^) and a T_2_*-weighted gradient-echo fast low-angle shot (FLASH) sequence (resolution = 500 × 500 × 500 μm^3^). CMBs were counted on T_2_*-weighted gradient-echo sequences by an experienced rater (S.J.V.V.). These lesions were defined as homogenous ovoid or round foci of low signal intensity. As *ex vivo* T_2_*-weighted scans are susceptible to air bubble artefacts, which can lead to false identification of CMBs, T_2_-weighted TSE scans were also used for comparison to help differentiate between CMBs and any remaining air bubbles.

For the targeted CAA case group, regions with high CMB burden were subsequently sampled as previously described.^3^ Briefly, each sample was submerged in Fomblin (Solvay Solexis, Thorofare, NJ, USA) in a 50 mL falcon tube. They were then scanned overnight using a custom-built solenoid coil and whole-body 7T MRI scanner (MAGNETOM; Siemens Healthineers). The protocol included a T_2_-weighted TSE sequence (resolution = 100 × 100 × 100 μm^3^) and a FLASH sequence (resolution = 75 × 75 × 75 μm^3^).^3^

### ARIA case

In addition to the abovementioned cases, one case who enrolled in the first clinical trial of active Aβ immunization for AD (AN1792; Elan Pharmaceuticals Inc.) and had documented ARIA-E during life was included as a positive control for inflammation and Aβ removal.^18^ This case provided consent for brain donation and ethical approval was obtained though the Southampton and South West Hampshire Local Research Ethics Committees (REC reference 075/03/w). One occipital slab was scanned with *ex vivo* 7T MRI and sections were sampled from a region with possible ARIA-H as described elsewhere.^19^ Serial sections were cut and stained as described below. Two haematoxylin and eosin (H&E) stained sections were screened for Grade 4 vessels.

### Tissue sampling

As mentioned above, for the targeted CAA case group, two cases with severe CAA were selected and temporo-occipital regions with high CMB burden were identified on 3T MRI, sampled and additionally scanned at 7T MRI prior to sectioning.^3^ For the five consecutive CAA cases, samples were taken from a prespecified regions of the frontal, parietal, temporal and occipital lobes as previously described.^3^ All samples were subsequently dehydrated, embedded in paraffin and cut into 6 μm thick serial sections with a microtome. For each sample, H&E staining was performed using standard histology protocols. Bright field immunohistochemistry against Aβ (mouse, catalog # M0872; Agilent Technologies, Santa Clara, CA, USA; 1:200), glial fibrillary acidic protein (GFAP) (rabbit, catalog # G9269; Sigma, St Louis, MO, USA; 1:1000), cluster of differentiation 68 (CD68; mouse, catalog # M0814; Agilent Technologies; 1:500), smooth muscle actin (SMA; ​mouse, catalog # M0851; Agilent Technologies; 1:250) and fibrin(ogen) (rabbit, catalog # A0080; Agilent Technologies; 1:500) was performed. The protocol for CD68 was implemented on a fully automated stainer (Bond Rx, Leica), and all other stains were performed manually following a standardized immunohistochemistry protocol.

Double fluorescent immunohistochemistry was performed against fibrin(ogen) (rabbit, catalog # A0080; Agilent Technologies; 1:500) and GFAP (mouse, catolog # G3893; Sigma; 1:1000) following a standardized protocol. Alexa Fluor™ 488 (1:500, Abcam catalog # ab150077) and 594 (1:500, Invitrogen catalog # A11005) IgG (H + L) cross-adsorbed secondary antibodies were used to tag fibrin(ogen) and GFAP, respectively.

Negative controls were performed for all stains listed above by omitting the primary antibody which showed no immunopositivity.

### Histopathological image analysis

Digital bright field and fluorescent microscopic images of the sections were obtained with the NanoZoomer Digital Pathology-HT scanner (C9600-12; Hamamatsu Photonics, Hamamatsu, Japan) using a 20x objective. The viewing platform NDP-View (v2.7.25) was used to analyse the digital images.

From each case, 1–2 sections were assessed, and cortical vessels were selected for analysis based on Aβ stains alone (blinded to other stains). Vessels were selected based on approximate vessel grade, with up to ∼20 vessels of each grade in each section as described below. Cortical vessels ≥20 μm in diameter were selected (to exclude capillaries) and all leptomeningeal vessels were excluded. Vessels associated with CMBs (defined as presence of perivascular haemosiderin deposits) were also excluded from analysis.

**Figure 1:**
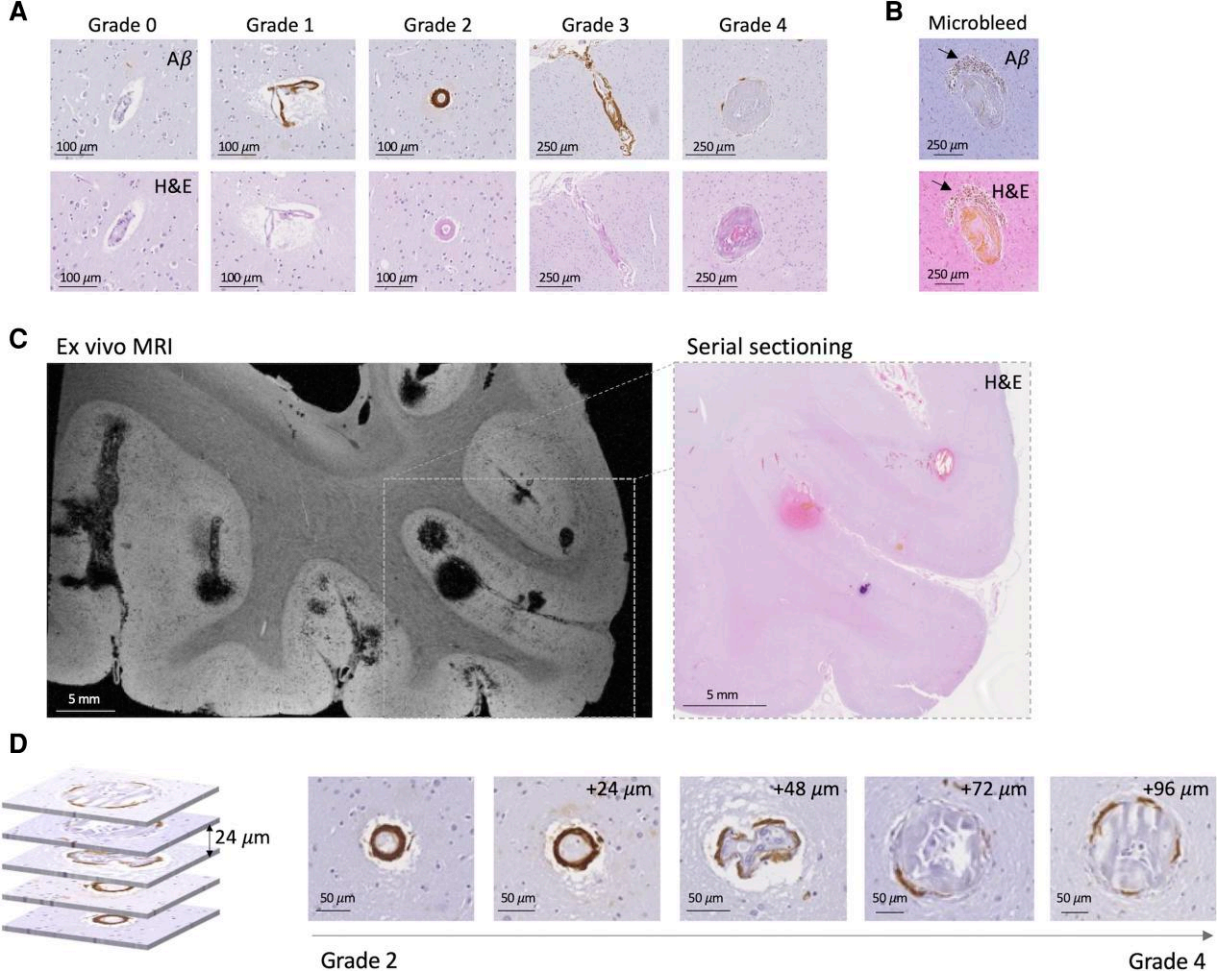
**Vessel grading scale and *ex vivo* MRI-guided serial sectioning in cases with definite CAA and microbleeds.** (**A**) Examples of representative Grades 0–4 vessels. (**B**) Example of representative CMB with perivascular hemosiderin deposits (arrows) (adapted with permission from van Veluw *et al*.^3^). Note the absence of Aβ in the vessel wall. (**C**) Left: *ex vivo* MRI T_2_*-weighted image with targeted region selected for serial sectioning and histopathology, as described previously.^3^ Right: H&E-stained section from selected region. (**D**) Multiple regions along a single vessel that transitions from Grades 2 to 4 along an ∼100 µm vessel segment.

Vessels were then independently graded using adjacent Aβ and H&E-stained sections by two trained raters (M.G.K. and I.Y.). The Greenberg and Vonsattel scale was used as follows: Grade 0—no Aβ deposition; Grade 1—patchy Aβ deposition, otherwise normal-appearing vessel wall; Grade 2—circumferential Aβ deposition; Grade 3—>50% splitting of the vessel wall, also known as ‘vessel-in-vessel’ or ‘lumen within lumen’ appearance; Grade 4—thickened vessel wall, evidence of fibrinoid necrosis defined as vessel walls with eosinophilic infiltrate with disrupted cytoarchitecture (Fig. 1).^15^ Inter-rater reliability was almost perfect (kappa = 0.93) and final vessel grades were determined in a consensus meeting. SMA stains were also used to eliminate veins from further analysis. Deidentified vessels were rated as arterioles versus venules by two independent raters (M.G.K. and I.Y.). When SMA stains demonstrated a lack of contractile smooth muscle cells, H&E stains were additionally used to assess vessel wall thickness and vessel lumen diameter to differentiate between arterioles and venules. Inter-rater reliability was substantial (kappa = 0.71) and final vessel classifications were also determined in a consensus meeting.

Vessel walls were classified as fibrin positive or negative by two independent raters blinded to all other stains (M.G.K. and I.Y.). Vessels were rated as positive when clear immunoreactivity against fibrin was apparent within the vessel wall, but not when immunoreactivity was only apparent within the lumen as previously described.^5^ Inter-rater reliability was excellent (kappa = 0.81) and final vessel fibrin ratings were determined at a consensus meeting. Vessels were additionally rated for SMA coverage using SMA stains with a scoring scale as follows: (3) complete SMA coverage, (2) circumferential SMA but incomplete vessel wall thickness, (1) non-circumferential and (0) no SMA coverage. Each vessel was rated by two independent raters (M.G.K. and I.Y.), blinded to all other stains with excellent inter-rater reliability (kappa = 0.81); final scores were determined in a consensus meeting.

Sholl analysis was performed to assess local inflammatory and BBB leakage markers surrounding the selected vessels. Each vessel was identified on fibrin, GFAP and CD68 stains, exported at a standardized magnification, and then covered by an opaque mask in Paint v21H1. At least 1 week thereafter, analysis was performed on deidentified images using a previously developed in-house interface in MeVisLab.^4^ Each vessel was outlined, and markers were manually placed on cells that stained positive for fibrin, GFAP and CD68, respectively, by a trained grader (I.Y.). Positive cells within 50 μm of the pial surface were excluded. The tissue surrounding each vessel was divided into 10 adjacent concentric shells measuring 50 μm in width each. For each shell, the density of positive cells was calculated in MeVisLab. To determine inter-rater reliability, two vessels were selected at random from a consecutive case and the densities of fibrin-, GFAP- and CD68-positive cells were calculated in the innermost shell by two experienced raters (I.Y. and M.G.K.). The intraclass correlation coefficient was 0.85, demonstrating good reliability.

### Statistical analysis

Statistical tests were performed comparing the numbers of reactive astrocytes, activated microglia and fibrin-positive cells surrounding individual vessels in the targeted cases using Kruskal–Wallis tests with *post hoc* pairwise comparisons using Mann–Whitney U-tests with *P*-value adjustment (Bonferroni). These tests were performed assuming vessel independence, a limitation of this portion of the analysis.

In the consecutive cases, for the analysis of BBB leakage and smooth muscle cell scores as well as reactive astrocytes and activated microglia in the innermost shells, Friedman *χ*^2^ tests were performed with *post hoc* pairwise comparisons using Conover’s test with *P*-value adjustment (Benjamini–Hochberg). A simple linear regression was performed to assess the association between CMBs and Grades 3 and 4 vessels.

All *P*-values are two-tailed and a threshold of *α* < 0.05 was used to determine statistical significance. Analyses were performed in Prism 9 and R.

### Data availability

Histopathological image analysis data for each individual vessel (including Vonsattel grade, fibrin and SMA ratings and Sholl analyses) are included in the Supplementary Tables. Additional data are available from the corresponding author upon reasonable request.

## Results

We hypothesized that by selecting regions from cases with severe CAA and multiple CMBs, we would enrich our samples with advanced grade vessels. Therefore, we used *ex vivo* MRI of brains from two cases (one female/one male) with confirmed severe CAA to select regions with a high microbleed burden (Table 1). We then performed serial sectioning and histopathology of these regions (Fig. 1, see Materials and methods for further details), as reported previously.^3^ These cases are referred to as targeted CAA cases. Within selected sections (total number of sections = 3), vessels were rated from Grades 0 to 4 (see Materials and methods, Fig. 1A), using a previously validated scale shown to correlate with bleed propensity.^6^ Both Grades 3 and 4 vessels demonstrate evidence of vascular remodelling. Grade 3 vessels have circumferential ‘cracking’ of the vessel wall, while Grade 4 vessels closely resemble the rupture site of vessels associated with microbleeds (Fig. 1B), including features of decreased Aβ coverage and vessel wall remodelling.^3^

### Tracing Grade 4 vessels through serial sectioning

All Grade 4 vessels identified in sections from these targeted CAA cases (*n* = 12 Grade 4 vessels in two cases) were manually traced along their course through serial sectioning. All Grade 4 vessels that could be traced were observed to ‘transition’ into Grade 2 segments (11/12 vessels; one vessel could not be reliably traced), suggesting that lower grade vessels may progress locally to more advanced vessel pathologies (Fig. 1D). Notably Grade 2 vessels have circumferential Aβ deposition while Grade 4 vessels have minimal to no Aβ, suggesting inhomogeneous initial deposition of Aβ and/or Aβ removal as vessels progress to Grade 4.

### BBB leakage observed at all vessel grades

**Figure 2:**
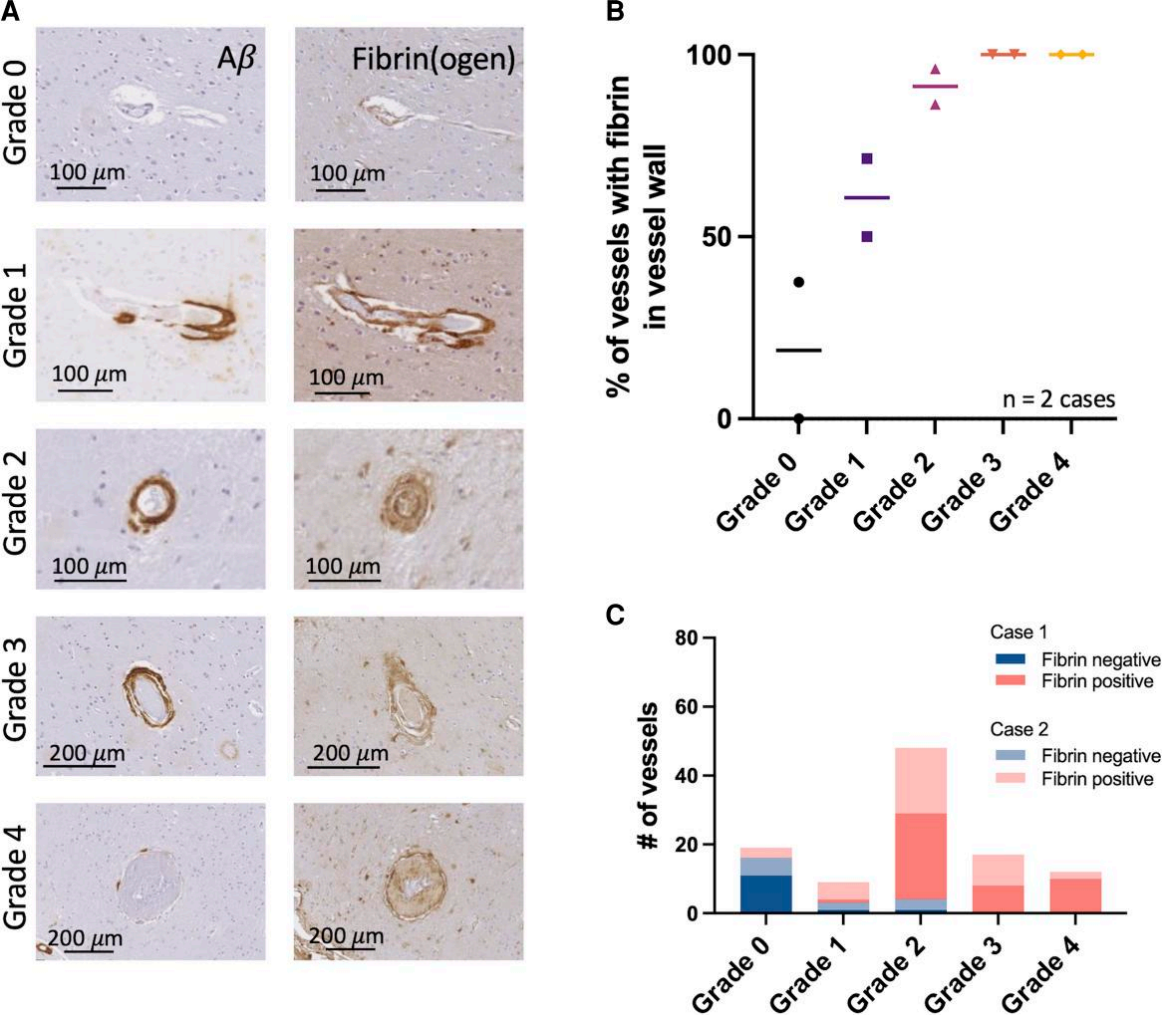
**Blood–brain barrier leakage can be observed starting in lower vessel grades.** (**A**) Aβ and fibrin(ogen) stains of representative examples of Grades 0–4 vessels. (**B**) Per cent of vessels within each vessel grade with evidence of fibrin(ogen) within their vessel wall across temporo-occipital sections from two ‘targeted’ cases with CAA (regions targeted based on high regional CMB burden). (**C**) Summary of fibrin vessel wall ratings in all vessels studied across two targeted cases with CAA. Number of vessels (Cases 1 and 2): Grade 0 = 11, 8; Grade 1 = 2, 7; Grade 2 = 26, 22; Grade 3 = 8, 9; Grade 4 = 10, 2.

Evidence of BBB leakage in the form of fibrin positivity in the wall of arterioles was observed in all vessel grades in targeted CAA cases, and the per cent of vessels with BBB leakage increased with higher vessel grades from 18.8% in Grade 0 to 100% in Grade 4 vessels (Fig. 2). Of note, the per cent of Grade 0 vessels (no Aβ deposition) with evidence of BBB leakage is similar to previously published findings of per cent fibrin positivity in control brain tissue.^5^ By Grade 2, over 90% of vessels identified were fibrin-positive, which was consistent across both targeted cases. Additionally, fibrin-positive cells were observed surrounding higher grade vessels with grade-dependent differences in the number of fibrin-positive cells up to ∼400 μm from each vessel (Supplementary Fig. 1A). The density of fibrin-positive cells in the innermost shell surrounding each vessel (1–50 µm from the vessel edge) increased significantly with vessel grade (Supplementary Fig. 1B; Kruskal–Wallis test, *P* < 0.0001). The finding of fibrin-positive perivascular cells suggests fibrin(ogen) extravasation from arterioles and subsequent cellular uptake. These cells appeared to be predominantly reactive astrocytes (Supplementary Fig. 1C).

### Perivascular inflammation observed predominantly in advanced grade vessels

**Figure 3:**
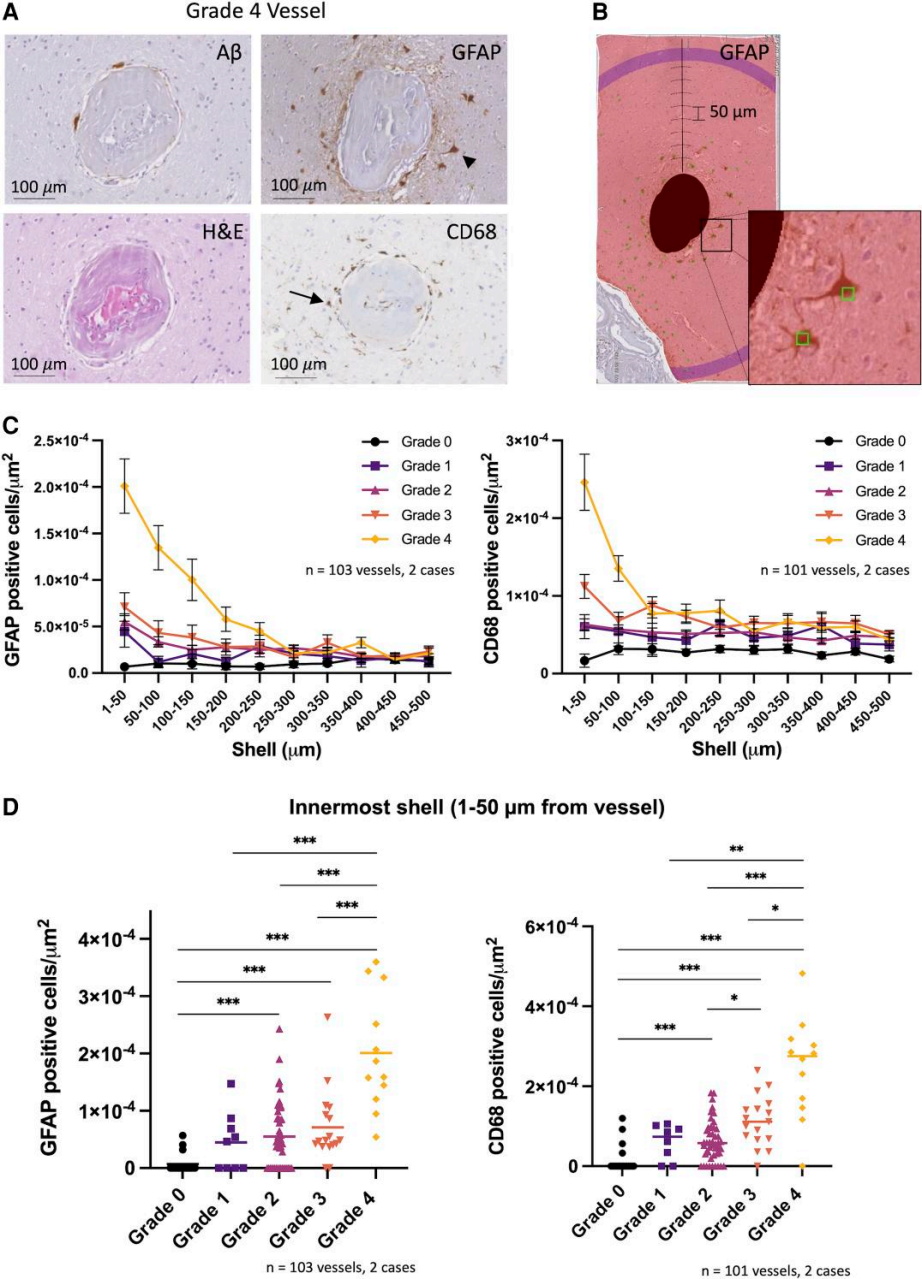
**Perivascular inflammation is observed predominantly around advanced grade vessels.** (**A**) Aβ, H&E, GFAP and CD68 stains of a representative Grade 4 vessel. Arrowhead and arrow point to examples of a reactive astrocyte and activated microglial cell respectively. (**B**) Example of Sholl analysis within the MeVisLab software environment for GFAP-positive cells surrounding a vessel. Masked vessel from **A** is shown with successive 50 µm shells and GFAP-positive cells manually annotated (square markers). Inset displays two marked GFAP-positive cells. (**C**) Density of GFAP-positive (left) and CD68-positive (right) cells in successive shells surrounding vessels from two targeted cases with CAA (mean ± SEM). (**D**) Density of GFAP-positive (left) and CD68-positive (right) cells in innermost shell (1–50 µm from vessel) shown for each vessel analysed (median shown). *P < 0.05, **P < 0.01, ***P < 0.005 (after Bonferroni correction). Kruskal–Wallis test, *P* < 0.0001 for both GFAP- and CD68-positive cells. *Post hoc* pairwise comparisons shown using Mann–Whitney U-test with Bonferroni *P*-value adjustment. Included in GFAP analysis, number of vessels (Cases 1 and 2): Grade 0 = 11, 8; Grade 1 = 2, 7; Grade 2 = 24, 22; Grade 3 = 8, 9; Grade 4 = 10, 2. Included in CD68 analysis, number of vessels (Cases 1 and 2): Grade 0 = 10, 8; Grade 1 = 1, 7; Grade 2 = 24, 22; Grade 3 = 8, 9; Grade 4 = 10, 2.

Perivascular inflammation, quantified as the density of reactive astrocytes (GFAP-positive) and activated microglia (CD68-positive) in shells surrounding each vessel, was observed to increase with increasing vessel grade. These effects were local and predominantly observed in the immediate perivascular area (Fig. 3C). Grade-dependent differences in the number of GFAP- and CD68-positive cells were observed up to ∼250–300 μm from each vessel. Focusing on the innermost shell surrounding each vessel (1–50 µm from the vessel edge), the density of GFAP- and CD68-positive cells increased significantly with vessel grade (Fig. 3D; Kruskal–Wallis test, *P* < 0.0001 for both GFAP- and CD68-positive cells). Of note, all Grade 4 vessels studied had evidence of perivascular inflammatory cells.

### Remodelled blood vessels are associated with higher numbers of microbleeds

**Figure 4:**
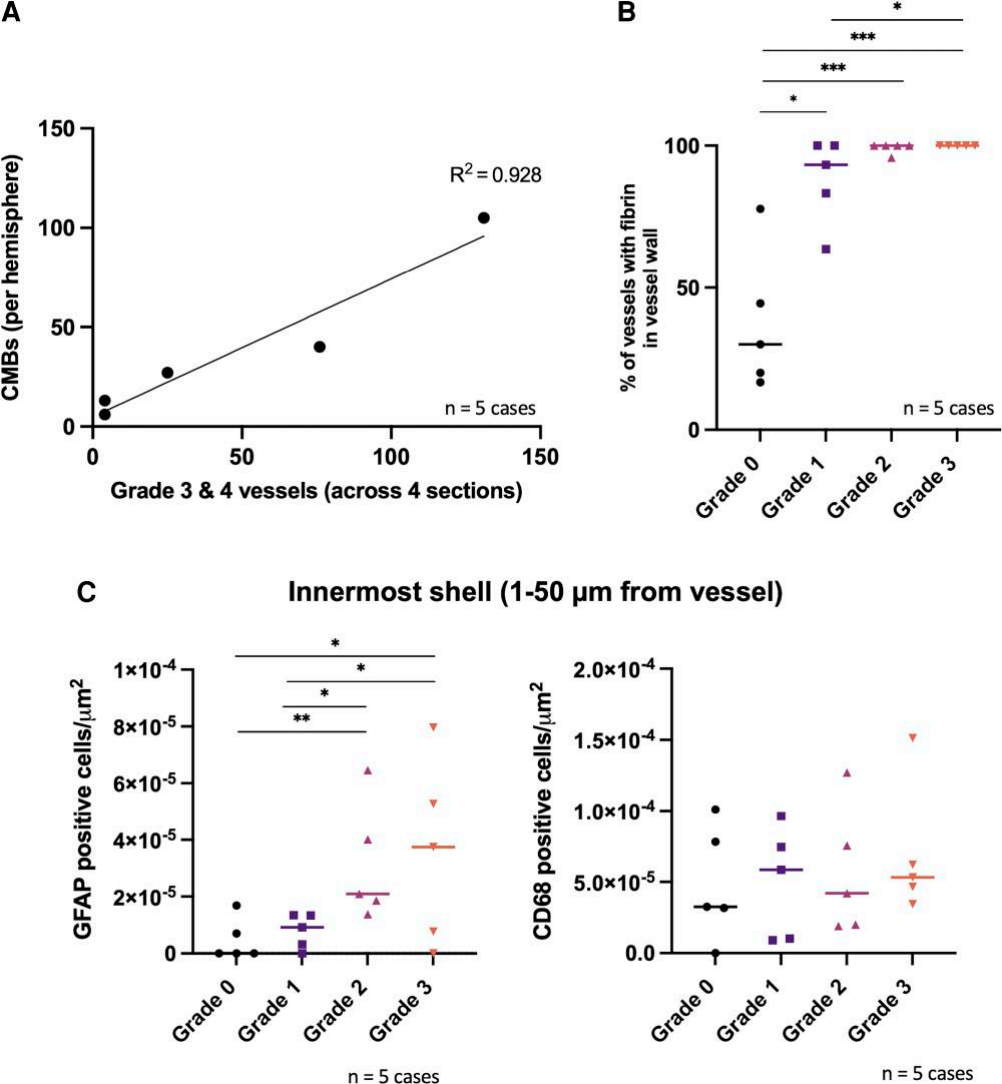
**Advanced grade vessels are associated with higher numbers of microbleeds as well as blood–brain barrier leakage and perivascular inflammation in consecutive CAA cases.** (**A**) Number of CMBs in each hemisphere as compared to the total number of Grades 3 and 4 vessels across four sections taken from standardized regions (frontal, parietal, temporal and occipital) from five consecutive cases with CAA. (**B**) Per cent of vessels within each vessel grade with evidence of fibrin(ogen) within their vessel wall across five consecutive cases with CAA (median shown) in the occipital cortex. Friedman *χ*^2^ (d.f. = 3) = 10.357, *P* = 0.016 with a large estimated effect size (Kendall’s *W* = 0.69). *Post hoc* pairwise comparisons shown using Conover’s test with *P*-value adjustment (Benjamini–Hochberg). Number of vessels (Cases 3–7): Grade 0 = 5, 10, 6, 9, 9; Grade 1 = 7, 15, 7, 11, 6; Grade 2 = 24, 30, 36, 31, 23; Grade 3 = 22, 4, 3, 1, 7. (**C**) Density of GFAP-positive cells (left) and CD68-positive cells (right) in the innermost shell surrounding each vessel (1–50 µm from vessel) (median shown). *P < 0.05, ***P < 0.005. For GFAP: Friedman *χ*^2^ (d.f. = 3) = 9.813, *P* = 0.020 with a large estimated effect size (Kendall’s *W* = 0.65). *Post hoc* pairwise comparisons shown using Conover’s test with *P*-value adjustment (Benjamini–Hochberg). For CD68: Friedman *χ*^2^ (d.f. = 3) = 1.56, *P* = 0.669) and the estimated effect size was small (Kendall’s *W* = 0.10). Included in GFAP analysis, number of vessels (Cases 3–7): Grade 0 = 5, 10, 6, 9, 9; Grade 1 = 7, 15, 7, 11, 6; Grade 2 = 24, 29, 35, 30, 22; Grade 3 = 22, 4, 3, 1, 7. Included in CD68 analysis, number of vessels (Cases 3–7): Grade 0 = 5, 9, 5, 6, 7; Grade 1 = 6, 13, 7, 11, 3; Grade 2 = 23, 30, 31, 28, 22; Grade 3 = 22, 4, 3, 1, 7.

To assess the relationship between CMB burden and remodelled vessels, we analysed sections from five additional CAA cases with varying degrees of CAA severity. We selected the five most recent brain donations in our MGH autopsy cohort and therefore refer to these as ‘consecutive’ CAA cases. All Grades 3 and 4 vessels were counted in individual sections taken from four standardized regions (frontal, parietal, temporal and occipital) across each hemisphere (total of four sections and one hemisphere per case analysed). We found a strong linear relationship between the number of Grades 3 and 4 vessels in these four standardized sections and the number of CMBs found on *ex vivo* MRI of that same hemisphere (Fig. 4A; simple linear regression, *R*^2^ = 0.928, *P*-value = 0.0083).

Grade 4 vessels were only observed in two of these cases in parietal sections (Cases 3 and 7), consistent with prior reports that Grade 4 vessels are associated with regions and cases with higher haemorrhagic burden.^15^

### BBB leakage and perivascular inflammation observed in consecutive CAA cases

To determine if our findings of BBB leakage and perivascular inflammation observed in targeted CAA cases are also observed in CAA cases with varying degrees of CAA severity, we performed a similar analysis to that performed in targeted CAA cases in individual occipital sections from five consecutive cases (one female/four males, Table 1). Similar to the targeted CAA cases, we observed BBB leakage at all vessel grades and found that the per cent of fibrin-positive vessels was significantly higher with higher vessel grade [Fig. 4A; Friedman *χ*^2^ (d.f. = 3) = 10.357, *P* = 0.016 with a large estimated effect size (Kendall’s coefficient of concordance (*W*) = 0.69)]. *Post hoc* pairwise comparisons showed statistically significant differences between vessel Grades 0 and 1 (*P* = 0.044), 0 and 2 (*P* = 0.0032), 0 and 3 (*P* = 0.0020) and 1 and 3 (*P* = 0.044).

Additionally, the density of reactive astrocytes surrounding each vessel was observed to increase with vessel grade, and these effects were most prominent in the innermost shell surrounding each vessel (Supplementary Fig. 2). In the innermost shell surrounding each vessel, there were statistically significant differences across vessel grade [Friedman *χ*^2^ (d.f. = 3) = 9.81, *P* = 0.020] with a large estimated effect size (Kendall’s *W* = 0.65). *Post hoc* pairwise comparisons showed statistically significant differences between vessel Grades 0 and 2 (*P* = 0.005), 0 and 3 (*P* = 0.010), 1 and 2 (*P* = 0.010) and 1 and 3 (*P* = 0.049). When comparing the density of CD68-positive cells in the innermost shell across vessel grades in the five consecutive CAA cases, there was no statistically significant difference [Friedman *χ*^2^ (d.f. = 3) = 1.56, *P* = 0.669] and the estimated effect size was small (Kendall’s *W* = 0.1). Of note, there was more variability in CD68-positive cell density across consecutive cases than in the targeted CAA cases (particularly with respect to the Grade 3 vessels). Additionally, there were limited numbers of Grade 3 vessels observed in the consecutive cases. For reference, representative Grade 3 vessels from each studied case are displayed in Supplementary Fig. 3.

### Grade 4 vessel identified in confirmed ARIA-E case

**Figure 5:**
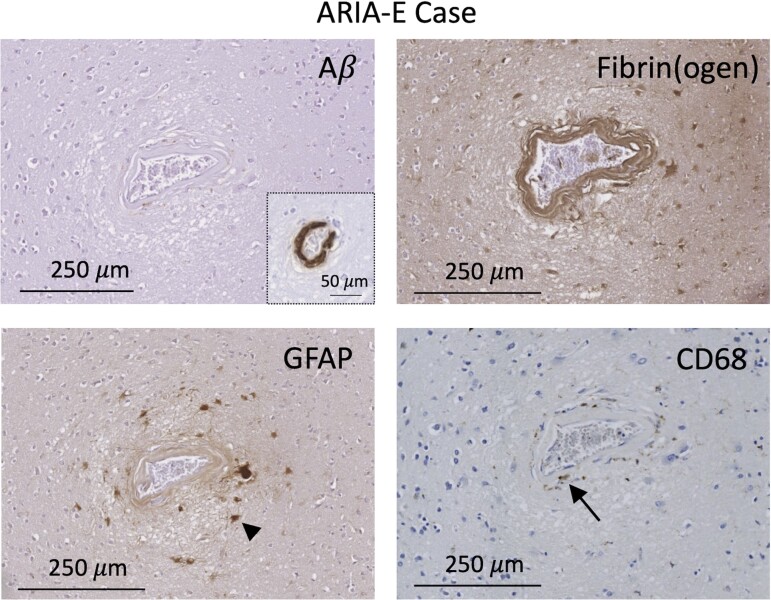
**Grade 4 vessel from case with known ARIA-E during life.** Adjacent Aβ, fibrin(ogen), GFAP and CD68 sections are shown. Vessel with no vascular Aβ, evidence of fibrin within the vessel wall and perivascular reactive astrocytes and activated microglia. Aβ inset displays Aβ deposition in a region of this vessel remote from the site of remodelling. Arrowhead and arrow point to examples of a reactive astrocyte and activated microglial cell, respectively.

A Grade 4 vessel with similar findings (no Aβ deposition, evidence of BBB leakage and perivascular inflammation) was identified in a case with Alzheimer’s disease who received active immunization (AN1792; Elan Pharmaceuticals Inc.) and developed ARIA-E during life (Fig. 5),^18^ suggesting potential shared vascular pathophysiology. Of note, Aβ was observed in the otherwise normal-appearing wall of this vessel remote from the site of remodelling.

### Contractile smooth muscle cell loss is observed at lower vessel grades

**Figure 6:**
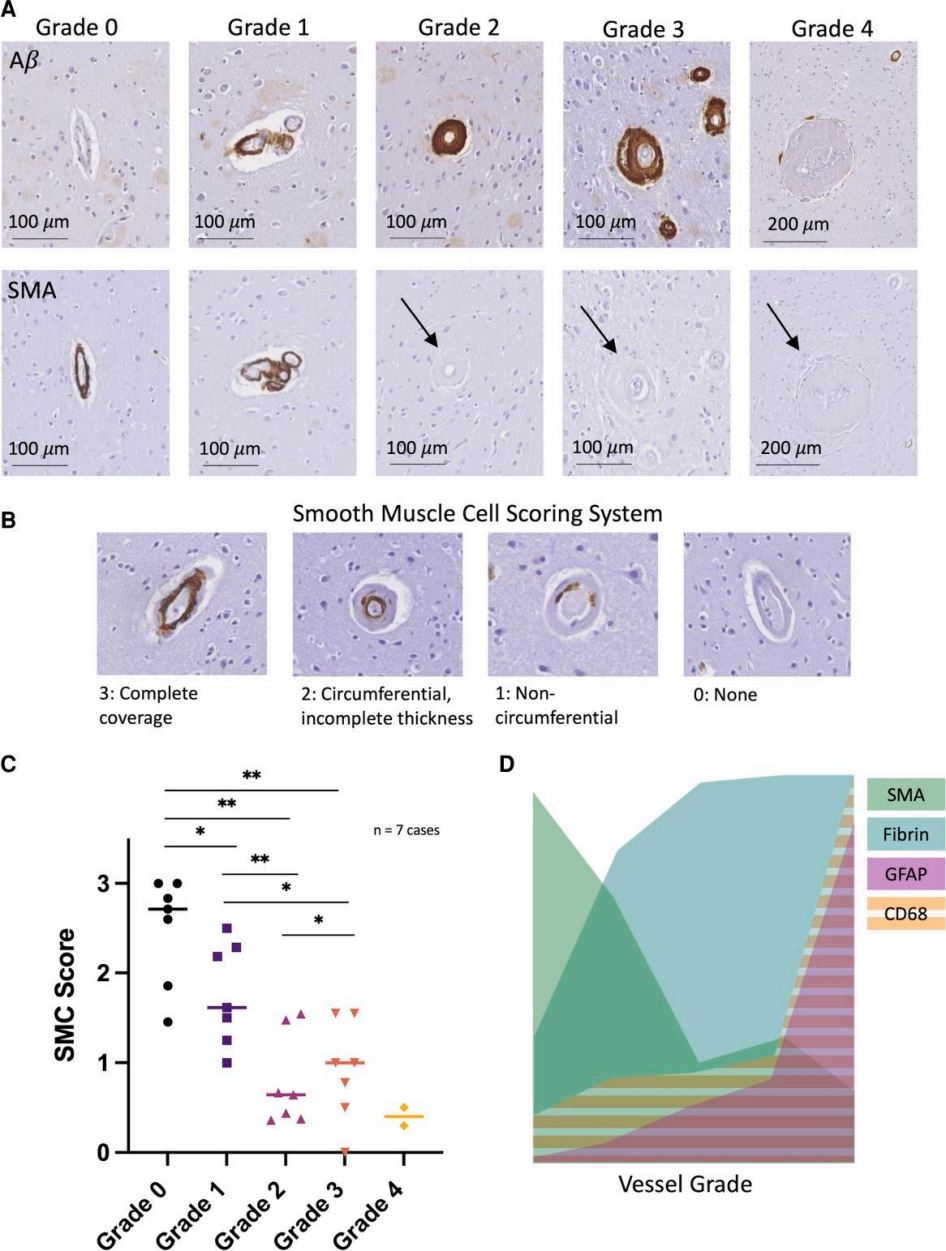
**Smooth muscle actin loss is observed starting in lower vessel grades.** (**A**) Aβ and SMA stains shown for representative Grades 0–4 vessels. Arrows point to vessel locations on SMA stain for Grades 2–4 vessels. (**B**) In-house developed smooth muscle cell scoring system. (**C**) Smooth muscle cell (SMC) score for each vessel grade across seven cases with CAA (two targeted cases and five consecutive cases, medians shown). (**D**) Overlay of SMA, fibrin, GFAP and CD68 pooled data across vessel Grades 0–4 for seven cases with CAA (two targeted cases and five consecutive cases). *P < 0.05, **P < 0.01. Friedman *χ*^2^ (d.f. = 3) = 15.514, *P* = 0.0014) with a large estimated effect size (Kendall’s *W* = 0.74). *Post hoc* pairwise comparisons shown using Conover’s test with *P*-value adjustment (Benjamini–Hochberg). Number of vessels (Cases 1–7): Grade 0 = 11, 7, 5, 7, 5, 6, 7; Grade 1 = 2, 7, 4, 13, 7, 11, 3; Grade 2 = 25, 21, 23, 25, 32, 28, 23; Grade 3 = 8, 9, 20, 2, 3, 1, 7; Grade 4 = 10, 2.

Because the loss of contractile smooth muscle cells may be part of the remodelling process as vessels progress from lower to more advanced grades, we assessed SMA across vessel grades in both targeted and consecutive CAA cases (Fig. 6). We developed a smooth muscle cell grading system to assess coverage (see Materials and methods, Fig. 6B). We observed overall lower SMA coverage with increasing vessel grade, starting at Grade 1 (Fig. 6C). These decreases in coverage were statistically significant across vessel grade [Friedman *χ*^2^ (d.f. = 3) = 15.514, *P* = 0.0014] with a large, estimated effect size (Kendall’s *W* = 0.74). *Post hoc* pairwise comparisons showed statistically significant differences in smooth muscle cell score between Grades 0 and 1 (*P* = 0.012), 0 and 2 (*P* = 2.6E − 6), 0 and 3 (*P* = 8.7E − 5), 1 and 2 (*P* = 0.00036) and 1 and 3 (*P* = 0.024). Interestingly, slightly higher SMA coverage was observed in Grade 3 compared with Grade 2 vessels (*P* = 0.047).

## Discussion

Through a detailed histopathological analysis of cases with CAA, this study demonstrated that advanced grade vessels are consistently associated with BBB leakage and perivascular inflammation. Additionally, higher numbers of remodelled advanced grade vessels (Grades 3 and 4) are associated with a higher CMB burden. Vessels with remodelling in the form of fibrinoid necrosis, i.e. Grade 4 vessels, closely resemble vessel segments associated with CMBs in recent studies^3^ and have previously been associated with lobar ICH.^6,15^ Grade 4 vessels were predominantly observed in targeted brain tissue, consistent with prior findings that these vessels are relatively rare and specific to cases and regions with higher haemorrhage burden.^15^

This study further investigated vascular remodelling in CAA through the assessment of vessels with varying grades of CAA pathology and found that both BBB leakage and the loss of contractile smooth muscle cells occurred already by vessel Grade 1. As such, these significant changes were observed even in Grade 1 vessels which have only patchy Aβ deposition with otherwise ‘normal’ appearing vessel walls on H&E. In fact, there appears to be little change in the percentage of vessels with evidence of BBB leakage or contractile smooth muscle cell coverage between Grades 2, 3 and 4, with the exception that Grade 3 vessels have significantly more contractile smooth muscle cell coverage than Grade 2 vessels. The difference between Grades 2 and 3 vessels may indicate Grade 3 vessels may follow a different path to vessel remodelling with more contractile smooth muscle cell preservation (and/or angiogenesis) in Grade 3 vessels than Grades 2 and 4 vessels. Prior work has shown increased matrix metalloproteinase-2 expression in Grades 2–4 vessels,^20^ a potential contributor to the early development of BBB leakage. The finding of early contractile smooth muscle cell loss is consistent with prior studies that have demonstrated impairments in vascular reactivity (which relies on smooth muscle cell contractility) in patients with a form of hereditary CAA (hereditary cerebral haemorrhage with amyloidosis-Dutch type) prior to the development of symptomatic haemorrhage or cognitive changes.^21^

By comparison, we observed that perivascular inflammation occurs predominantly surrounding advanced grade vessels, most consistently and markedly surrounding Grade 4 vessels (Fig. 6D), but also occurring to a lesser extent around lower grade vessels. Both reactive astrocytes and activated microglia were observed, and numbers of perivascular reactive astrocytes were more consistently observed to increase with higher vessel grades. Reactive astrocytes were observed to have a stronger spatial relationship to the vessels studied, and other factors affecting brain inflammation may have affected the numbers of activated microglia observed leading to increased variability. Notably, Grade 3 remodelled vessels had more variable levels of associated perivascular inflammation in consecutive cases, but this analysis is limited by the relatively low numbers of Grade 3 vessels that were observed in the consecutive cases. Inflammation may not in all cases be an essential driver of the vessel ‘cracking’ form of vascular remodelling. One possibility is that compensatory processes which may partially preserve vessel integrity/function, such as angiogenesis, may lead to a Grade 3 ‘vessel-in-vessel’ appearance. Prior work has demonstrated increased angiogenic factors such as VEGF and upregulation of pro-angiogenic genes in Alzheimer’s disease,^22–24^ and pathological angiogenesis may play an important role in vascular remodelling in CAA.^25^ Of note, the amount of adaptive angiogenesis in CAA and Alzheimer’s disease is likely limited; overall impairments in angiogenesis in Alzheimer’s disease have been reported and Aβ itself may have an antiangiogenic effect.^26,27^

The interactions between BBB leakage and perivascular inflammation in CAA and Alzheimer’s disease are complex and likely bidirectional. Aβ is a known trigger for both local inflammation^28–30^ and BBB leakage.^31^ Chronic inflammation leads to BBB leakage and conversely BBB leakage triggers perivascular inflammation,^31–34^ and recent work has suggested that fibrinogen itself is a trigger for microglial activation.^35^ Notably, other components of the neurovascular unit including pericytes and oligodendrocytes are also affected by fibrinogen deposition,^16^ and these may be important contributors to microvascular dysfunction in CAA. Our data suggest that, at the level of single-vessel segments, BBB leakage appears to occur at an earlier stage of CAA pathology than perivascular inflammation as measured by the presence of activated microglia and reactive astrocytes. These results suggest that early BBB leakage in CAA may in fact be a driver of perivascular inflammation.

The close association between Grades 2 and 4 vessel segments observed through individual vessel tracing suggests that Grade 2 vessel segments may progress directly to Grade 4. However, Grade 4 vessel segments have significantly less Aβ within their vessel walls than Grade 2 vessel segments. This potential loss or removal of vascular Aβ may in part relate to the observed perivascular inflammation. Prior studies have shown the capacity of reactive astrocytes and activated microglia to internalize and degrade Aβ.^17^ While the inflammation observed in these cases appears to be largely limited to innate immune responses, there may be shared mechanisms with the conditions CAA-ri and ARIA in which inflammation has been linked to both local Aβ removal and haemorrhage,^11–14^ supported by evidence of a Grade 4 vessel observed in a single section in a case with known ARIA-E during life.

This study has several limitations. First, the nature of *ex vivo* studies is inherently cross-sectional. As such, we are unable to truly track the sequence of events that occurred in the brain tissue and may even ‘miss’ important events affecting the fate of a vessel. Further studies, including those using animal models of CAA, are necessary to further delineate these events. Additionally, this study examined a limited number of markers of BBB leakage and neuroinflammation with a focus on demonstrating that both BBB leakage and perivascular inflammation occur consistently in CAA. While fibrin(ogen) staining of the vessel wall is indicative of BBB leakage, future work dedicated to exploring the microarchitecture of vessel walls affected by CAA with a focus on direct assessment of the presence of tight junctions and other cell types that modulate the BBB may yield important insights. Likewise, exploration of additional markers of neuroinflammation may further delineate potential mechanisms. Finally, the sample size of this study was small, included predominantly male subjects, and consisted of autopsy cases from a select group of individuals who chose to donate their brains; therefore, these findings may not be generalizable to the larger population of CAA patients.

## Conclusions

These cross-sectional neuropathological findings in cases with CAA suggest that BBB leakage and contractile smooth muscle cell loss may precede vascular remodelling (in the form of vessel wall splitting and fibrinoid necrosis) in individual cortical arterioles affected by CAA. The finding of perivascular inflammation around high-grade vessels suggests that this pathway may be in part mediated by inflammation and that BBB leakage may be an important trigger for perivascular inflammation and possible Aβ removal. BBB leakage and perivascular inflammation therefore represent potential future therapeutic targets for haemorrhage prevention in CAA.

## Supplementary Material

fcac245_Supplementary_Data

## Abbreviations

Aβ =: amyloid-β
ARIA =: amyloid-related imaging abnormalities; BBB = blood–brain barrier
CAA =: cerebral amyloid angiopathy
CAA-ri =: CAA-related inflammation
CD68 =: cluster of differentiation 68
CMBs =: cerebral microbleeds
cSS =: cortical superficial siderosis
GFAP =: glial fibrillary acidic protein
H&E =: haematoxylin and eosin
ICH =: intracerebral haemorrhage
SMA =: smooth muscle actin
