# Supplementary material for: Blood–brain barrier leakage and perivascular inflammation in cerebral amyloid angiopathy
Source: Brain Commun. 2022 Sep 26;4(5):fcac245. doi: 10.1093/braincomms/fcac245 (PMC9576155; doi:10.1093/braincomms/fcac245)
Supplement: fcac245_Supplementary_Data [file fcac245_supplementary_data.zip › Supplementary_figures.pdf]

Supplementary Information

Supplementary Figure 1

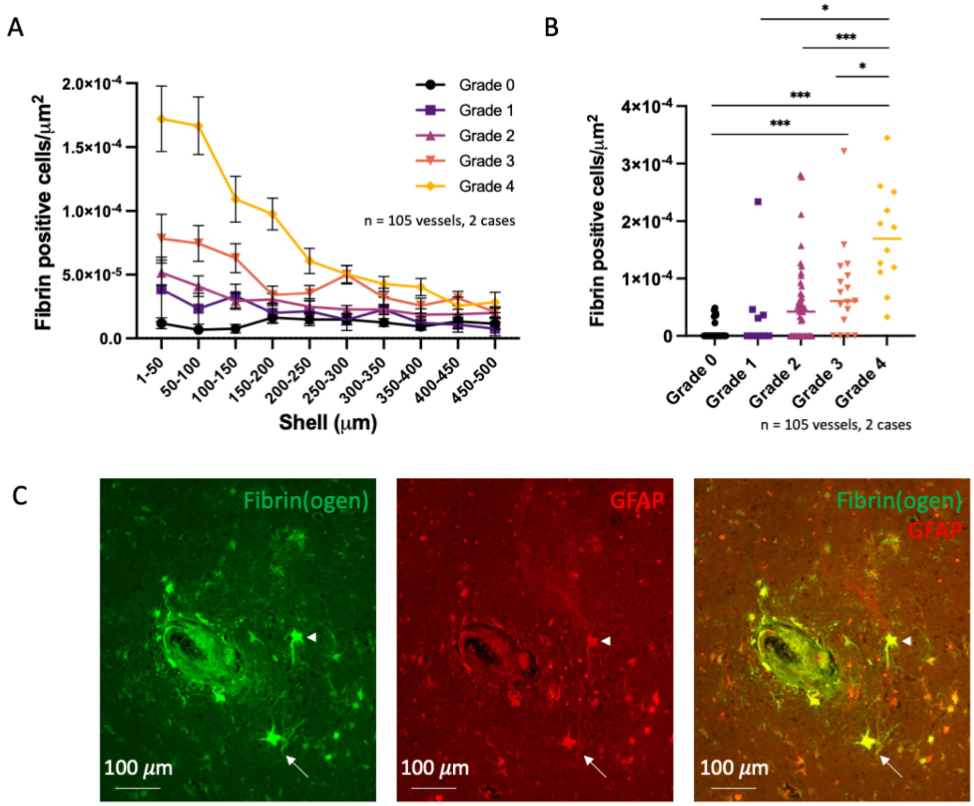

Fibrin positive cells are observed primarily surrounding advanced grade vessels and include reactive astrocytes. (**A**) Density of fibrin positive cells in successive shells surrounding vessels from 2 targeted cases with CAA (mean +/- SEM). (**B**) Density of fibrin positive cells in innermost shell (1-50 μm from vessel) shown for each vessel analyzed (median shown). Kruskal-Wallis test, $p < 0.0001$. Post-hoc pairwise comparisons shown using Mann-Whitney U tests with Bonferroni p-value adjustment. *$p < 0.05$, **$p<0.01$, ***$p<0.005$ (after Bonferroni correction). # of vessels (case 1, case 2): Grade 0 = 11, 8; Grade 1 = 2, 7; Grade 2 = 26, 22; Grade 3 = 8, 9; Grade 4 = 10, 2. (**C**) Fibrin(ogen) and GFAP double immunofluorescence stain of a representative grade 4 vessel. Arrow and arrowhead indicate fibrin positive reactive astrocytes.

Supplementary Figure 2

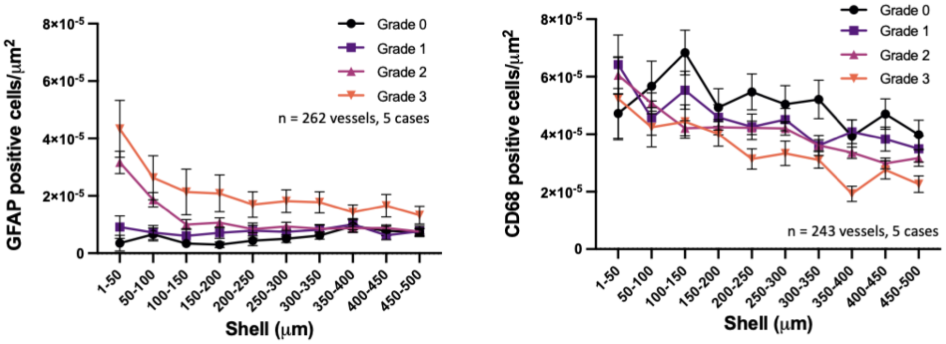

Perivascular inflammation in consecutive CAA cases. Density of GFAP positive (left) and CD68 positive (right) cells in successive shells surrounding vessels from 5 consecutive cases with CAA (mean +/- SEM). Included in GFAP analysis, # of vessels (cases 3 – 7): Grade 0 = 5, 10, 6, 9, 9; Grade 1 = 7, 15, 7, 11, 6; Grade 2 = 24, 29, 35, 30, 22; Grade 3 = 22, 4, 3, 1, 7. Included in CD68 analysis, # of vessels (cases 3 – 7): Grade 0 = 5, 9, 5, 6, 7; Grade 1 = 6, 13, 7, 11, 3; Grade 2 = 23, 30, 31, 28, 22; Grade 3 = 22, 4, 3, 1, 7.

Supplementary Figure 3

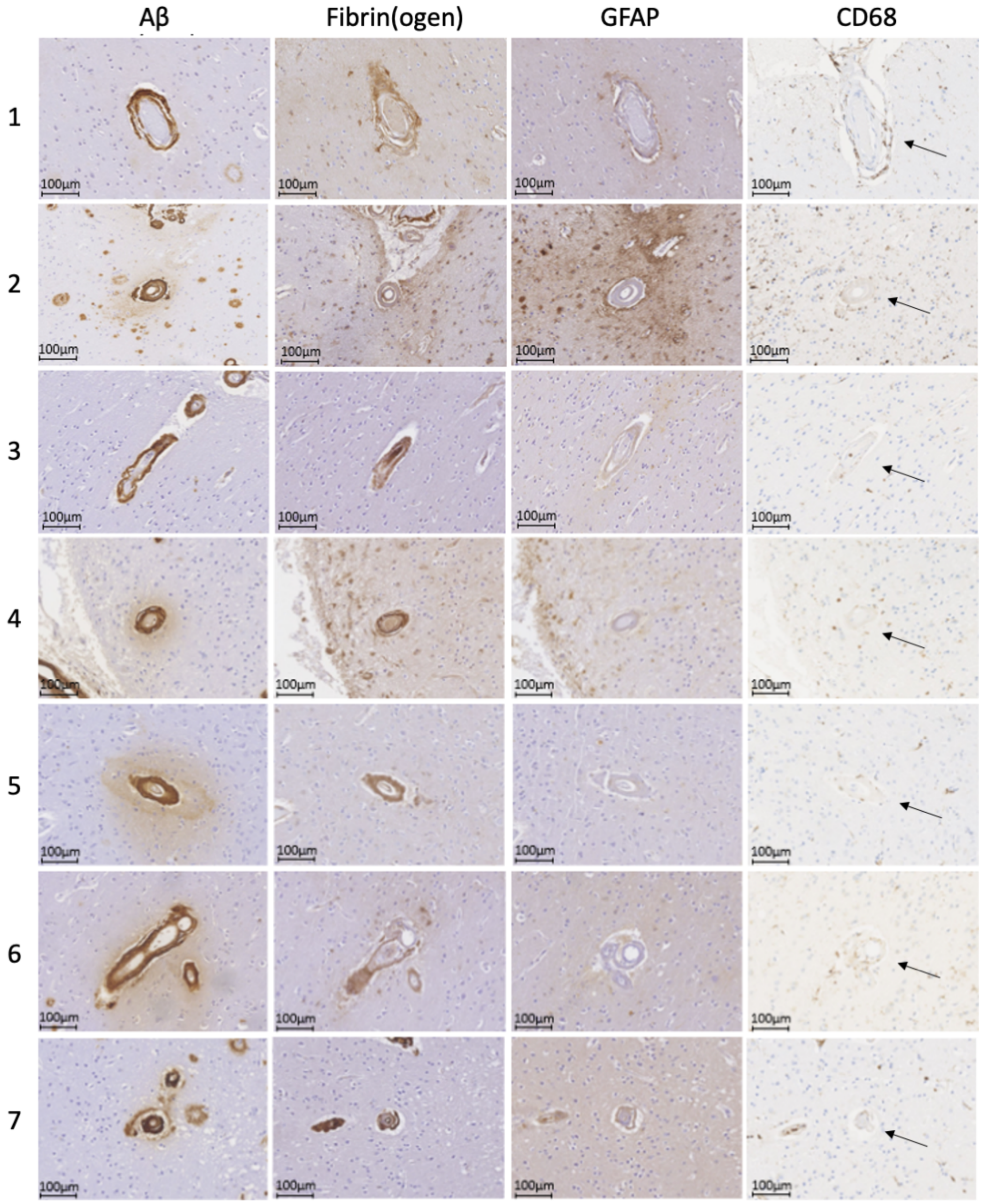

Perivascular inflammation surrounding Grade 3 vessels is variable across cases. Adjacent Aβ, fibrin(ogen), GFAP, and CD68 stains for representative vessels from all 7 cases (2 targeted and 5 consecutive) shown. All vessel walls are fibrin(ogen) positive, suggesting BBB leakage. Arrows point to vessel locations on CD68 stain.
